# Self-Referential Cognition and Empathy in Autism

**DOI:** 10.1371/journal.pone.0000883

**Published:** 2007-09-12

**Authors:** Michael V. Lombardo, Jennifer L. Barnes, Sally J. Wheelwright, Simon Baron-Cohen

**Affiliations:** Autism Research Centre, Department of Psychiatry, University of Cambridge, Cambridge, United Kingdom; Claremont Graduate University, United States of America

## Abstract

**Background:**

Individuals with autism spectrum conditions (ASC) have profound impairments in the interpersonal social domain, but it is unclear if individuals with ASC also have impairments in the intrapersonal self-referential domain. We aimed to evaluate across several well validated measures in both domains, whether both self-referential cognition and empathy are impaired in ASC and whether these two domains are related to each other.

**Methodology/Principal Findings:**

Thirty adults aged 19-45, with Asperger Syndrome or high-functioning autism and 30 age, sex, and IQ matched controls participated in the self-reference effect (SRE) paradigm. In the SRE paradigm, participants judged adjectives in relation to the self, a similar close other, a dissimilar non-close other, or for linguistic content. Recognition memory was later tested. After the SRE paradigm, several other complimentary self-referential cognitive measures were taken. Alexithymia and private self-consciousness were measured via self-report. Self-focused attention was measured on the Self-Focus Sentence Completion task. Empathy was measured with 3 self-report instruments and 1 performance measure of mentalizing (Eyes test). Self-reported autistic traits were also measured with the Autism Spectrum Quotient (AQ). Although individuals with ASC showed a significant SRE in memory, this bias was decreased compared to controls. Individuals with ASC also showed reduced memory for the self and a similar close other and also had concurrent impairments on measures of alexithymia, self-focused attention, and on all 4 empathy measures. Individual differences in self-referential cognition predicted mentalizing ability and self-reported autistic traits. More alexithymia and less self memory was predictive of larger mentalizing impairments and AQ scores regardless of diagnosis. In ASC, more self-focused attention is associated with better mentalizing ability and lower AQ scores, while in controls, more self-focused attention is associated with decreased mentalizing ability and higher AQ scores. Increasing private self-consciousness also predicted better mentalizing ability, but only for individuals with ASC.

**Conclusions/Significance:**

We conclude that individuals with ASC have broad impairments in both self-referential cognition and empathy. These two domains are also intrinsically linked and support predictions made by simulation theory. Our results also highlight a specific dysfunction in ASC within cortical midlines structures of the brain such as the medial prefrontal cortex.

## Introduction

Autism spectrum conditions (ASC) involve impairments in social functioning, language or communication, and the presence of stereotyped repetitive behaviors and/or highly restricted interests. Historically, the “self” has been integral in what it means to have autism. The term “autism” (derived from the Greek word “autos”, which literally means “self”) was first coined by Bleuler to characterize many of the social withdrawal symptoms exhibited by schizophrenics. Later, Kanner[1] applied this term to the children in his clinic whom he observed to be completely self-focused. Most recently, Frith[2] has developed a theoretical perspective that puts the self at the core of all the observed impairments and strengths in ASC. Frith's theory differs from Kanner in that, instead of viewing ASC as a syndrome of complete self-focus, it is viewed under the notion of an “absent self”.

The first reason for proposing an absent self comes from several observations that detail abnormalities in ASC due to weak central coherence[3], executive dysfunction[4], and varying degrees of mindblindness[5]. One commonality among all of these theories is a lack of what psychologists have historically called the “central executive”; that is, a lack of top-down control of bottom-up information processing in the brain[2]. Recent neuroimaging studies have confirmed that all of these abnormalities are due to deficient top down modulation of bottom up information processing by frontal regions of the brain[6], [7], [8], [9], [10], [11] and are concurrent with the notion of enhanced short range but diminished long range connectivity in the autistic brain [10], [11], [12], [13], [14], [15].

At the psychological level of analysis, the absent self theory also proposes that self-awareness is less developed in ASC. When individuals with ASC are asked to report on the content of randomly sampled daily experiences, their reports relied on physical descriptions of the moment rather than on their own mental and emotional states[16]. During structured interviews that elicit statements about various aspects of the self, adolescents with ASC gave fewer descriptions of themselves in social contexts[17]. Adults with ASC may also not show the typical facilitative effect of self-referential cognition on memory (the “self-reference effect in memory”)[18]. Individuals with ASC also report more difficulty with identifying and describing their own emotions; what is known clinically as “alexithymia”[19]. Atypical first person pronoun usage has also been well documented in ASC[1], [20], [21], [22], [23]. While older theories have related this abnormality to a form of echolalia[1] or to abnormalities in person deixis[22], normative studies in social psychology regard first person pronoun usage as an index of self-focused attention[24]. Thus, the available work implicates that self-referential cognition may be abnormal in ASC.

In the present study we aimed to further examine whether self-referential cognitive processing is abnormal in ASC and to examine the interaction of self-referential cognition and empathizing. First, we tested whether individuals with ASC would show the typical self-reference effect in memory (SRE)[25]. Typically developing adults show a robust SRE when self-referential information processing is compared to information processing in relation to other people or to semantic manipulations[26]. The SRE is also positively related to trait self-consciousness[27] and works through enhancing organizational and elaborative cognitive processing of information[28], [29]. To compliment the SRE paradigm, we included several other well validated self-report and performance measures of self-referential cognition. Furthermore, since both cognitive and affective empathy impairments have been well documented in ASC[30], [31], [32], [33], several self-report and performance measures of empathy were included. We predicted that across all empathy and self-referential cognitive measures, individuals with ASC would show concurrent impairments.

**Figure 1:**
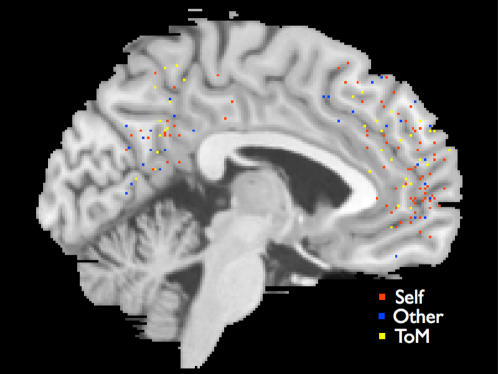
Image showing the overlap in peaks of activation from studies of self-referential cognition, other-referential cognition, and theory of mind within the medial prefrontal cortex and posterior cingulate/precuneus. Boundaries are 16mm from within midline. All peaks are taken from exemplary studies in the literature. Brain is depicted on a representative sagittal slice of the Montreal Neurological Institute (MNI) template (x = −2).

Within the typically developing brain, both self-referential cognitive processing and mentalizing are consistently localized to neural processing in cortical midline structures (CMS) of the brain such as the medial prefrontal cortex (MPFC) and posterior cingulate/precuneus (PC)[34], [35], [36], [37], [38], [39] (see Figure 1). However, mentalizing does not selectively recruit CMS regions of the brain in ASC[6], [40], [41], [42], [43]. This observation has led some to theorize that CMS regions of the brain play a role in using simulation as a mentalizing strategy[44], [45]. Simulation theory suggests that one way we know about the psychological characteristics of others is by using information about ourselves to construct a default model of how other minds work. To the extent that someone is similar to oneself, one can then evaluate how much of one's own characteristics should be imputed onto others[46], [47], [48]. Consistent with predictions made by simulation theory, we predicted that individuals with ASC would show decreased memory for themselves and for similar close others, but show no differences in memory for a dissimilar non-close other or for non-social information. Furthermore, to corroborate the link between self-referential cognition and mentalizing we also predicted that there would be significant individual differences in how self-referential cognitive processing is related to mentalizing in both ASC and normally developing controls.

## Methods

### Participants

All participants gave informed consent to participate in this study in accordance with the University of Cambridge Psychology Research Ethics Committee. There were 23 males and 7 females aged 19–45 that had an official diagnosis by internationally accepted criteria[49], [50] of either high-functioning autism (HFA; n = 4) or Asperger Syndrome (AS; n = 26). Controls consisted of 23 males and 7 females who were pairwise matched on age and gender and had no known psychiatric, developmental, or neurological disorders. All participants completed the Autism Spectrum Quotient (AQ)[51] and the ASC group scored higher than controls on the AQ (p<0.001). All participants scored in the normal range on the Wechsler Abbreviated Scale of Intelligence (WASI)[52] and there were no statistically significant group differences on verbal, performance, or full scale IQ (all p>0.90). See Table 1 for participant characteristics.

**Table 1:** Participant characteristics and manipulation checks on the SRE paradigm.

	Control	ASC	t value	p value	Cohen's d
**Age**	29.93 (7.83)	29.13 (7.40)	0.407	p = 0.686	0.11
**VIQ**	116.47 (8.65)	116.13 (12.81)	0.118	p = 0.906	0.03
**PIQ**	114.43 (10.08)	114.17 (14.21)	0.052	p = 0.958	0.01
**FIQ**	117.10 (8.65)	117.23 (13.11)	−0.047	p = 0.963	0.01
**AQ**	16.50 (6.38)	33.93 (7.89)	−9.408	p<0.001	2.47
**Potter Familiarity**	4.53 (3.42)	3.70 (3.21)	0.973	p = 0.334	0.25
**Self-Friend Similarity**	4.13 (0.97)	4.13 (0.94)	0.000	p = 1.000	0.00
**Self-Potter Similarity**	2.63 (1.13)	2.23 (1.10)	1.387	p = 0.171	0.36
**Self-Friend Closeness**	4.80 (1.49)	4.70 (1.58)	0.252	p = 0.802	0.07
**Self-Potter Closeness**	2.70 (1.37)	2.13 (1.41)	1.581	p = 0.119	0.42
**Friend Likeability**	5.33 (0.61)	5.07 (1.17)	1.106	p = 0.273	0.29
**Potter Likeability**	4.07 (1.34)	4.23 (1.38)	−0.475	p = 0.637	0.12

Means and standard deviations (in parentheses) are shown along with corresponding t values, p values, and effect sizes (Cohen's d) for between group comparisons.

### Procedure and Measures

#### SRE Paradigm

The paradigm used to assess the SRE was a depth-of-processing paradigm[53]. During the encoding phase, participants judged trait adjectives in one of four ways. In the “self” condition, participants judged how descriptive the adjective was of themselves. In the “similar close other” condition, adjectives were judged on how descriptive it was of their best friend. In the “dissimilar non-close other” condition participants judged whether the adjective was descriptive of Harry Potter[54]. All of these judgments were made on a 6 point scale where 1 indicated “not at all descriptive” and 6 indicated “very descriptive”. Finally, in the non-social control condition, participants judged how many syllables each adjective contained (from 1–6). Each condition had 30 trials and all were presented in pseudorandom order. All adjectives were drawn from a previously validated and widely used set of trait adjectives[55]. Half the adjectives in each condition were positively valenced (e.g., inventive) while the other half were negatively valenced (e.g., messy). Among all conditions there were no differences in number of characters, syllables, valence, or frequency of the adjectives.

After encoding there was a 30 minute delay before the retrieval phase. Participants were completely unaware of the subsequent recognition memory task to follow. During this delay participants completed the performance section of the WASI. These tasks were non-verbal and were administered to keep the participant occupied during the delay period.

After the delay, participants were given a surprise recognition memory test. All 120 adjectives from encoding and 120 new distracter adjectives were presented in pseudorandom order. Participants judged their confidence in whether the adjective was “old” or “new”. Confidence judgments were made on a 1–6 scale where 6 was “definitely OLD” and 4 was “OLD, but kind of unsure”. Conversely, a 1 indicated that they were “definitely NEW” and a 3 was “NEW but kind of unsure”. This 6 point scale was used to force participants to make finer grained recognition judgments and also to investigate whether there were any differences in how each group used different confidence judgments. There were no group differences among judgments on each scale point within any of the conditions (all p>0.10). Therefore, we collapsed judgments 1–3 into “New” and 4–6 into “Old” judgments.

#### Manipulation Checks

Prior to testing, to ensure all participants were familiar with Harry Potter we set an inclusion criterion to have seen at least one Harry Potter film or to have read at least one book. We obtained a familiarity index for Harry Potter by adding up the number of movies seen and books read. Groups did not differ on this Harry Potter familiarity index (p = 0.334).

After the SRE paradigm participants were asked how similar and close they thought they were to their friend and to Harry Potter and how likeable they perceived their friend or Harry Potter to be. Similarity and likable judgments were rated on a 1–6 scale, where 1 was “not at all” and 6 was “very much”. For the closeness measure, we used the Inclusion of Other in Self scale (IOS)[56]. The IOS depicts closeness spatially with Venn diagrams of two circles. One circle is labeled “Self” and the other circle was either labeled “Friend” or “Harry Potter”. Participants are given 7 choices that vary in degree of overlap between the “Self” circle and the other circle. Larger numbers indicate judgments of feeling close and including another person within the self.

Confirming our similarity and closeness manipulations, within each group participants viewed themselves and their best friend to be closer and more similar than the Self-Harry Potter judgment. Both groups also liked their friend more than Harry Potter (all p<0.009). However, there were no differences between groups for either best friend or Harry Potter on similarity, closeness, or liking (all p>0.12). See Table 1.

#### Empathy Measures

The Empathy Quotient (EQ)[57], Interpersonal Reactivity Index (IRI)[58], and the Emotional Contagion Scale (ECS)[59] were our three self-report measures of empathy. The EQ was rescored into cognitive and affective empathy based on items identified from previous factor analyses[60]. On the IRI, our indices of cognitive and affective empathy were the perspective taking and empathic concern subscales respectively. The IRI also has two other subscales (Fantasy and Personal Distress), but these were not included as measures of cognitive or affective empathy. Finally, our performance measure of empathy, indexing mentalizing ability, was the Reading the Mind in the Eyes Test (Eyes)[61].

#### Self-Consciousness/Awareness Measures

Self-reported self-consciousness/awareness was measured with the Private Self-Consciousness Scale (PSCS)[62] and the Toronto Alexithymia Scale (TAS)[63]. We also included a performance measure of self-focused attention called the Self-Focus Sentence Completion task[64]. In this task, participants were given sentence stems that included a self-reference (e.g., *I* think…, If *I* had *my* way…). For each stem, we asked participants to complete the sentence in whatever way they liked. Self-focused attention (SFA) was the dependent variable and was computed automatically with the Linguistic Inquiry and Word Count program[65] as the percentage of first person pronouns used to complete the sentences (e.g., I, me, myself). This index has been used several times as a valid quantitative index of SFA[66], [67], [68].

### Statistical Analyses

To evaluate our main hypotheses within the SRE paradigm, we ran a repeated measures ANOVA with four within subject levels and group as the between subject variable. Our dependent variable was a standard measure of memory sensitivity (d') formulated as the standardized score of correctly remembered words minus the standardized score of false alarms. Because we hypothesized that groups would differ on Eyes test performance and because it was theoretically relevant to test the effects of the SRE paradigm independent of mentalizing ability, we ran another repeated measures ANCOVA with Eyes test included as a covariate. Second, we computed two different SRE variables. The first SRE was a difference score for self versus friend (d' Self-d' Friend) while the second SRE was the difference score for self versus Harry Potter (d' Self-d' Harry Potter). Larger scores on these SRE variables indicate larger biases for self-referentially encoded information compared to other-referentially encoded information. One sample t-tests in each group were ran to evaluate whether each SRE variable was significantly different from 0. Independent samples t-tests were also ran to determine whether groups differed on the SRE variables. Third, we examined whether groups differed on empathy and self-consciousness measures using independent samples t-tests. Measures of effect size (Cohen's d) were also computed for each t-test comparison.

To examine individual differences between self-referential cognition and mentalizing we ran multiple regression analyses with Eyes test as the dependent variable and measures of self-referential cognition (self memory, TAS, PSCS, SFA) as independent variables. In our regression models we determined statistically whether relationships between mentalizing and self-referential cognition differed as a function of diagnosis using procedures outlined by Aiken and West[69]. In all, four regression models were evaluated (one model for each self-referential independent variable) and each model had the main effects of the self independent variable and a dummy variable for group entered on the first step. In the second step of our regression model, we entered the product vector of the interaction between the self independent variable and group membership (Group x Self Independent Variable). If the product vector interaction term was significant, this meant that the relationship between self-referential cognition and mentalizing was statistically different between groups (e.g., slopes were not parallel between groups). If this occurred, we conducted a simple linear regression, but this time separately on each group to discern how the relationships varied by diagnosis. However, if the product vector was not significant, this meant that there was no difference in the relationship between self-referential cognition and mentalizing as a function of diagnosis (e.g., slopes were parallel between groups). Therefore, in this case we ran a simple linear regression model but this time with the data collapsed across groups. The same procedures were used to examine the relationship between self-referential cognition and the AQ. In these analyses however, the AQ was the dependent variable.

## Results

**Figure 2:**
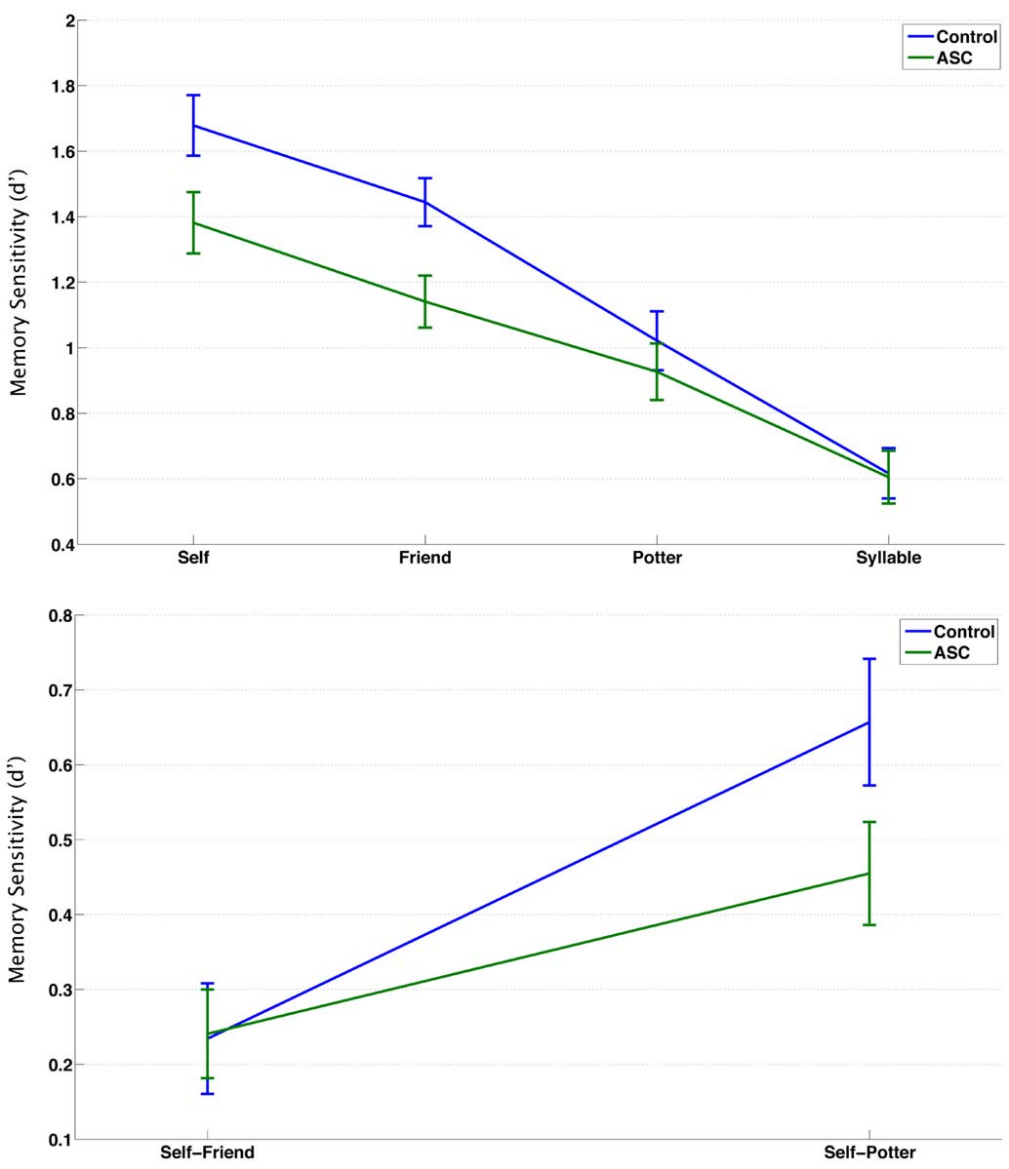
Line graph depicting recognition memory performance (top) and self-referential biases in memory (bottom) during the SRE paradigm. Bars indicate +/− one SEM.

Our first analysis looked at performance on the SRE paradigm. The repeated measures ANOVA showed an interaction effect between encoding level and group (F(3,58) = 3.599, p = 0.015). Therefore, we looked at the simple effects of encoding level within and between groups to discern the basis of the interaction. Consistent with our predictions, between group comparisons at each level of encoding showed that individuals with ASC were not impaired on memory for words previously judged on the basis of counting syllables (t(58) = 0.105, p = 0.916, Cohen's d = 0.03) or when judging Harry Potter's personality (t(58) = 0.716, p = 0.450, Cohen's d = 0.19). However, individuals with ASC had decreased memory for words previously judged in relation to themselves (t(58) = 2.265, p = 0.027, Cohen's d = 0.59) or their best friend (t(58) = 2.814, p = 0.007, Cohen's d = 0.73). See Table 2 and Figure 2.

**Table 2:** SRE paradigm data.

	Control	ASC	t value	p value	Cohen's d
**Self**	1.68 (0.51)	1.38 (0.51)	2.265	p = 0.027	0.59
**Friend**	1.44 (0.40)	1.14 (0.43)	2.814	p = 0.007	0.73
**Potter**	1.02 (0.49)	0.93 (0.47)	0.761	p = 0.450	0.19
**Syllable**	0.62 (0.42)	0.61 (0.44)	0.105	p = 0.916	0.03
**SRE (Self-Friend)**	0.23 (0.40)	0.24 (0.32)	−0.064	p = 0.949	0.02
**SRE (Self-Potter)**	0.66 (0.46)	0.45 (0.38)	1.858	p = 0.068	0.49

Means and standard deviations (in parentheses) for d' measures along with t values, p values, and effect sizes (Cohen's d) for between group comparisons.

Within each group there was a linear trend replicating the effect of depth of processing within the SRE paradigm[26]. The hierarchy of memory performance was Self>Friend>Potter>Syllable. Post-hoc Bonferroni corrected comparisons confirmed that all conditions for both groups were different from each other (all p<0.02). Furthermore, within each group, SREs were greater than zero for Self-Friend (Control: t(29) = 3.175, p = 0.004; ASC: t(29) = 4.068, p = 0.0003) and for Self-Harry Potter (Control: t(29) = 7.768, p<0.001; ASC: t(29) = 6.617, p<0.001). However, between groups, the SRE for Self-Harry Potter was larger among controls and approached statistical significance (t(58) = 1.858, p = 0.068, Cohen's d = 0.49) while the SRE for Self-Friend was not different between groups (t(58) = −0.064, p = 0.949, Cohen's d = 0.02). See Table 2 and Figure 2.

When Eyes test was included as a covariate, the interaction between group and encoding level approached statistical significance (F(3,57) = 2.471, p = 0.064). Testing between group differences, we found only memory performance for the best friend condition was different, with controls having better memory than ASC (t(57) = 2.001, p = 0.05, Cohen's d = 0.53). Within each group the hierarchy in memory performance still existed (Self>Friend>Other>Syllable; all p<0.02). The group difference for the SRE of Self-Harry Potter approached statistical significance, with controls having a large bias that ASC (t(57) = 1.710, p = 0.093, Cohen's d = 0.45) while no differences remained for the SRE of Self-Friend (t(57) = −0.015, p = 0.988, Cohen's d = 0.003).

In our next analysis we examined whether there were concurrent impairments in self-referential cognition and empathy in ASC. Individuals with ASC scored lower across all empathy measures (Cognitive EQ, Affective EQ, IRI Perspective Taking, IRI Empathic Concern, ECS, Eyes test; all p<0.05). On the IRI Fantasy subscale, individuals with ASC were also lower than controls (t(58) = 2.507, p = 0.015, Cohen's d = 0.66). However, on the IRI Personal Distress subscale, individuals with ASC scored higher (t(58) = −3.195, p = 0.002, Cohen's d = 0.84). Individuals with ASC showed no differences on the PSCS (t(58) = 0.561, p = 0.577, Cohen's d = 0.15), but did report more alexithymia on the TAS (t(58) = −5.315, p<0.001, Cohen's d = 1.40). Among the TAS subscales, individuals with ASC had more difficulty identifying and describing their own feelings (DIF, DDF) and also reported more externally oriented thinking (EOT) (all p<0.001). Individuals with ASC were also lower in SFA on the Self-Focus Sentence Completion test (t(58) = 2.136, p = 0.037, Cohen's d = 0.56). See Table 3.

**Table 3:** Empathy and self-consciousness/awareness data.

	Control	ASC	t value	p value	Cohen's d
**IRI EC**	18.93 (5.16)	15.83 (6.09)	2.127	p = 0.038	0.56
**IRI PT**	18.50 (5.30)	14.33 (5.49)	2.991	p = 0.004	0.79
**IRI FS**	17.77 (5.69)	13.87 (6.34)	2.507	p = 0.015	0.66
**IRI PD**	10.60 (4.00)	14.53 (5.42)	−3.195	p = 0.002	0.84
**ECS**	41.97 (7.99)	37.47 (8.16)	2.158	p = 0.035	0.57
**Cognitive EQ**	15.27 (5.25)	4.17 (3.81)	9.381	p<0.001	2.46
**Affective EQ**	14.47 (6.30)	5.87 (3.83)	6.388	p<0.001	1.68
**Eyes Test**	27.03 (3.90)	23.73 (6.67)	2.340	p = 0.023	0.61
**PSCS**	30.50 (4.16)	29.80 (5.42)	0.561	p = 0.577	0.15
**TAS**	41.97 (9.19)	58.37 (14.19)	−5.315	p<0.001	1.40
**DIF**	13.50 (4.82)	20.03 (6.70)	−4.337	p<0.001	1.14
**DDF**	11.10 (4.85)	16.87 (5.62)	−4.252	p<0.001	1.12
**EOT**	17.37 (4.16)	21.47 (4.90)	−3.493	p<0.001	0.92
**SFA**	0.11 (0.03)	0.09 (0.03)	2.136	p = 0.037	0.56

Means and standard deviations (in parentheses) along with t values, p values, and effect sizes (Cohen's d) for between group comparisons. EC, Empathic Concern; PT, Perspective Taking; FS, Fantasy; PD, Personal Distress; ECS, Emotional Contagion Scale; EQ, Empathy Quotient; PSCS, Private Self-Consciousness; TAS, Toronto Alexithymia Scale; DIF, Difficulty Identifying Feelings; DDF, Difficulty Describing Feelings; EOT, Externally Oriented Thinking; SFA, self-focused attention.

**Figure 3:**
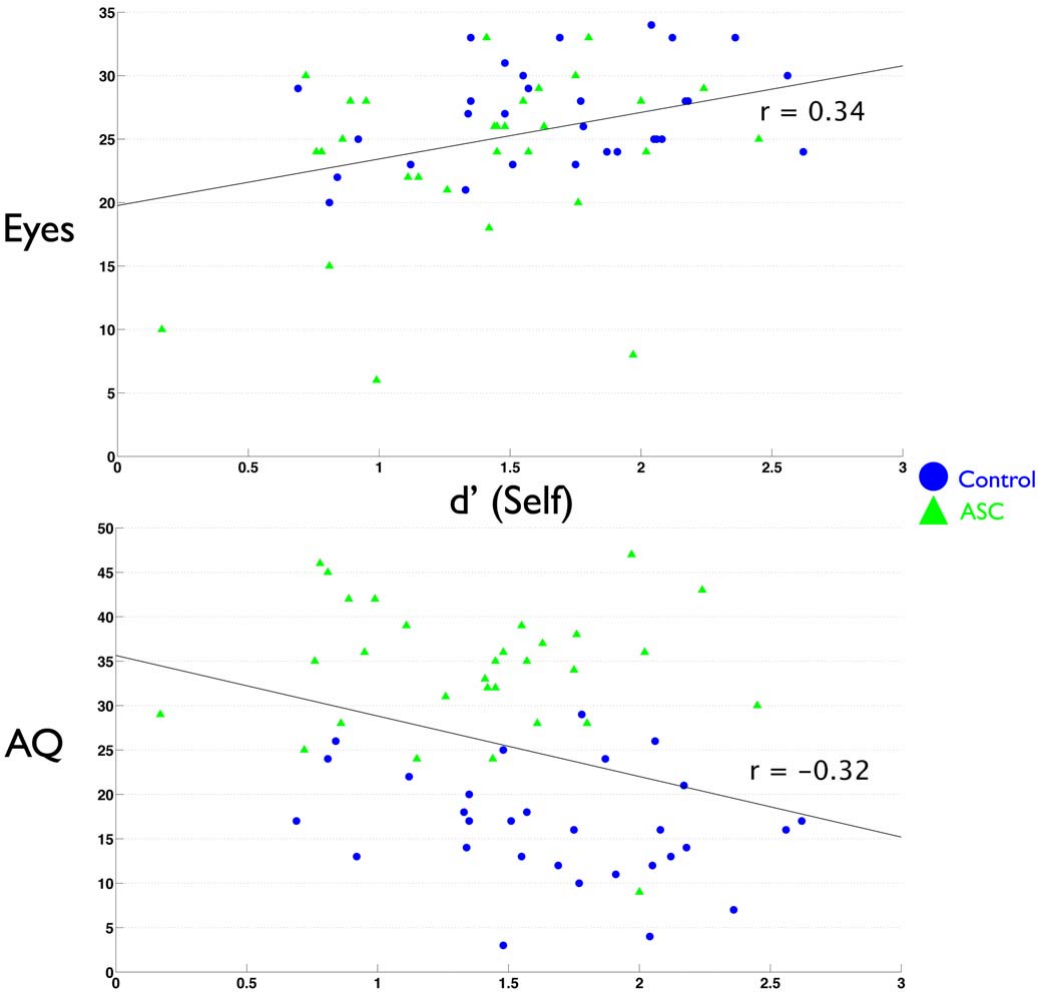
Scatterplots depicting the relationship between self memory and Eyes test (top) or AQ scores (bottom).

Next, we examined whether individual differences in self-referential cognition were related to mentalizing ability. No differences existed between groups in the relationship between Eyes test and self memory performance or TAS (indicated by a non-significant Group x Self interaction term in the regression model). Thus, since slopes were similar across groups, we collapsed the data across groups and looked at the effect of self-referential cognition on mentalizing. We found that regardless of diagnosis, self memory performance was positively related to Eyes test performance (r = 0.34, p = 0.007). Alexithymia was also negatively related to Eyes test performance (r = −0.43 p<0.001). Private self-consciousness and self focused attention however, showed differential relationships with the Eyes test that were dependent upon diagnosis. There was a significant interaction effect between how PSCS and diagnosis predicted Eyes performance (F change(1,56) = 4.917, p = 0.031). Inspecting the relationship PSCS had with the Eyes test separately within each group revealed that the interaction effect was driven by a strong positive correlation between PSCS and Eyes test performance in individuals with ASC (r = 0.50, p = 0.005) but not controls (r = −0.01, p = 0.951). Regarding the relationship that SFA had with Eyes test performance, we found another significant interaction effect between SFA and diagnosis (F change (1,56) = 6.627, p = 0.013). Upon looking at the relationship between SFA and Eyes test performance separately for each group, we found the interaction effect was driven by a negative relationship between SFA and Eyes test performance in the control group while individuals with ASC showed a positive relationship between SFA and the Eyes test. Both of these correlations approached statistical significance (Controls: r = −0.32, p = 0.08; ASC: r = 0.35, p = 0.06). See Figures 3–6.

**Figure 4 pone-0000883-g004:**
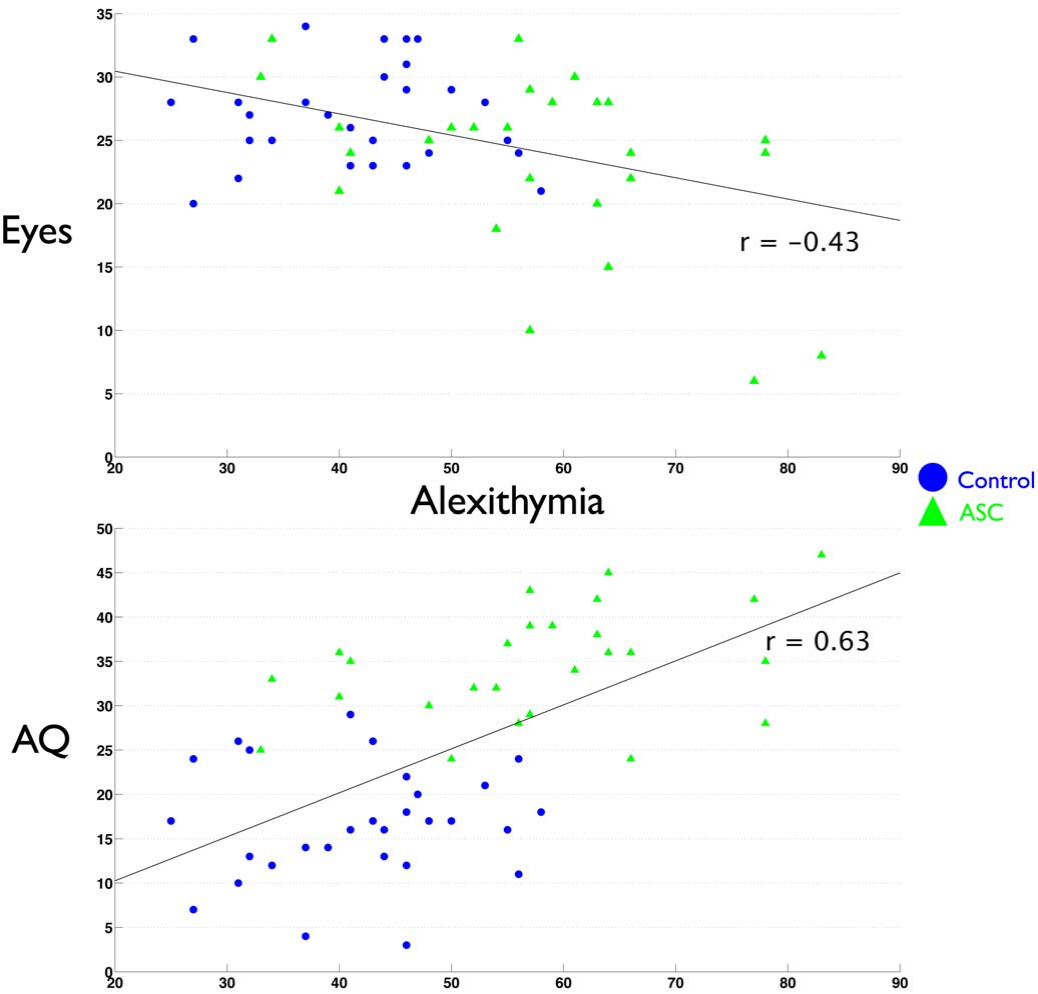
Scatterplots depicting the relationship between alexithymia and Eyes test (top) or AQ scores (bottom).

**Figure 5 pone-0000883-g005:**
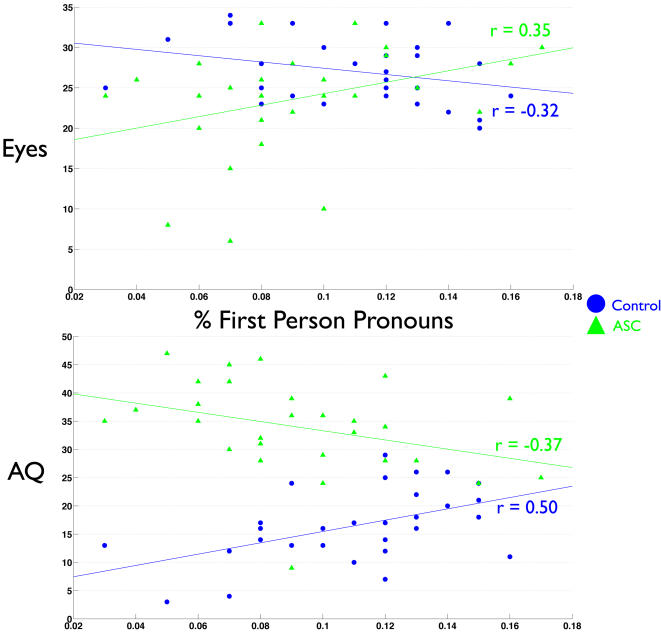
Scatterplots depicting the relationship between self-focused attention and Eyes test (top) and AQ scores (bottom). The index of self-focused attention is the percentage of first person pronouns used on the Self-Focus Sentence Completion test.

**Figure 6:**
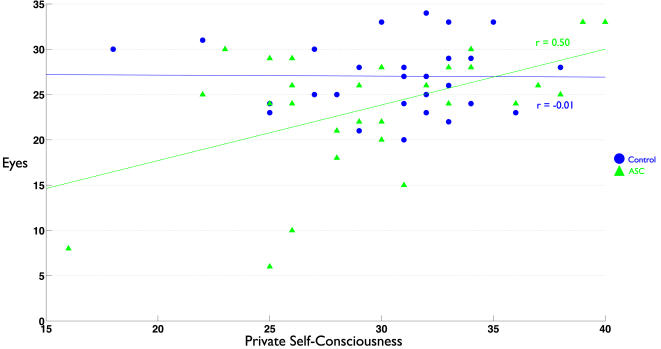
Scatterplot depicting the relationship between self-focused attention and Eyes test performance. The index of self-focused attention is a reflective form of self-focused attention as measured by the Private Self-Consciousness Scale.

In similar analyses, we looked at how self-referential cognition predicts scores on the AQ. There was no interaction effect among group and self-memory, TAS, or PSCS. Therefore, after collapsing across groups we found that increasing TAS scores predicted more autistic traits (r = 0.63, p<0.001) and that increasing self memory predicted less autistic traits (r = −0.32 p = 0.01). There was no relationship between PSCS and AQ scores. When modeling the relationship between SFA and AQ scores, there was a significant interaction effect between SFA and diagnosis (F change (1,56) = 12.38, p<0.001). Thus, we looked at the relationship of SFA on AQ scores separately for each group. Among controls, higher SFA predicts higher AQ scores (r = 0.50, p = 0.005). The opposite is true for individuals with ASC; higher SFA predicts lower AQ scores (r = −0.37, p<0.05). See Figures 3–6.

## Discussion

This study confirmed through multiple measures that individuals with ASC have difficulties in the self-referential cognitive domain. In the SRE paradigm, individuals with ASC show the typical SRE but to a reduced extent compared to age and IQ matched controls. People with ASC also showed reduced memory for themselves and for a similar close other but did not show any memory impairments for a dissimilar non-close other or for non-social memory. Thus, the only group differences in memory occurred for conditions where self information was to be remembered, or for information about others who significantly overlap with the self; similar close others.

Individuals with ASC are not however, completely impaired in self-referential information processing. We did not replicate the findings from the Toichi et al., study[18]. Unlike the Toichi study, which claimed that there was no SRE in ASC, we did find significant SREs in the ASC group. The differences in our study and the Toichi study are worth mentioning because they shed light on the differing findings. First, the Toichi study used a biased measure of memory performance in the percentage of words that were correctly recognized. We used a more unbiased measure of memory sensitivity (d') that corrects for the false alarm rate. Second, unlike the Toichi study, we included comparison conditions for thinking about other people. When self memory is compared to memory for others, both the control and ASC groups showed the typical SRE. SREs for self versus a similar close other are identical among the two groups. Given that there are SRE effects within the ASC group, it cannot be the case that people with ASC are not facilitated by self information.

Similar and close others have been hypothesized to overlap with the self. Several investigators have found effects supporting this hypothesized overlap and that the neural substrate involved in this processing may be MPFC[37], [44], [45], [48], [56], [70]. Thus, our comparison of an SRE between self and best friend may not be the best measure of a pure SRE. In fact, the self and best friend overlap quite a bit within our manipulation checks of closeness and similarity. Our best comparison of whether individuals really do exhibit a bias for self information is the SRE for self versus a dissimilar non-close other. When this comparison was investigated, individuals with ASC did show a trend for having smaller SREs than controls. This result brings up the idea that the impairment in using self information in ASC is one of *degree* and not *complete absence*. Further work at the neural level will be able to tell us more about the degree of impairment and the extent to which self- and other-referential cognitive processing overlap in the brains of people with ASC.

Our findings on several other measures of self-referential cognition also highlight its importance in ASC. Individuals with ASC report more alexithymia and are less self-focused but do not differ in private self-consciousness. Our findings extend on past work[16], [19] and show that deficits in self-referential cognition are not simply because of differences in the *amount* of self-reflection they engage in (as indexed by no group differences in PSCS and intact SREs), but has more to do with the *ease* of knowing about one's own inner emotional life. We mirror the conclusions of Berthoz and Hill[71], in that individuals with ASC can and do monitor their own inner states to some degree. However, it remains unclear what is the direction of the relationship between their known difficulties in understanding their own emotions and their attentional biases and future research should be directed at parsing apart this question.

We also showed in several ways that self-referential cognition and empathy are inextricably linked. First, our results within the SRE paradigm showed that mentalizing accounted specifically for self memory (indicated by the lack of a group difference in self memory after the Eyes test was included as a covariate) but not for memory in other-referential or non-social encoding conditions. Furthermore, some individual differences in self-referential cognition were related to mentalizing, regardless of whether an individual had a diagnosis. As self memory increased, mentalizing also increased and the endorsement of autistic traits decreased. The same pattern of individual differences (regardless of diagnosis) was manifest in the relationship between alexithymia and mentalizing. Here, more alexithymia is related to worse mentalizing and more endorsement of autistic traits.

Although we found some individual differences in self-referential cognition and mentalizing that were independent of diagnosis, we also found some very interesting and important relationships between self-referential cognition and mentalizing that depend upon diagnosis. In particular, self-focused attention and mentalizing were differentially related depending on diagnosis. ASC individuals who are more self-focused are better at mentalizing. However, among controls, being more self-focused predicted less mentalizing ability. A similar pattern existed with self-focused attention and endorsement of autistic traits. ASC individuals who are more self-focused report having less autistic traits. Among controls, those who are more self-focused report having more autistic traits. Another measure that showed a differential relationship based on diagnosis was the private self-consciousness scale. In the social psychological literature, private self-consciousness is believed to be a *reflective* form of self-focused attention[24] and is a good compliment to our performance measure of self-focused attention (the Self-Focus Sentence Completion task), which was more implicit and *non-reflective* in nature. On this measure of more reflective self-focused attention, a similar pattern emerged to that of implicit non-reflective self-focused attention. ASC individuals who report being higher in private self-consciousness are better at mentalizing. However, among controls private self-consciousness was not related to mentalizing. Because these results are correlational it is hard to tease apart why these relationships exist and further manipulations of self-focused attention will be needed to understand the mechanisms underlying these relationships. However, one finding is clear: being more self-focused appears to be beneficial for individuals with ASC. For now, we speculatively conclude that these findings might point to the idea that people with autism need to be more self-focused and have more metacognitive ability to accurately reflect on themselves in order to mentalize with others. If this is true, it would support the idea that simulation is a strategy that individuals with ASC could benefit from and may be helpful in informing novel treatments for more able high-functioning individuals on the autism spectrum.

Overall, our results implicate dysfunction within CMS regions of the brain in ASC. The concurrent impairments and relationships observed for both self-referential cognition and empathy are striking given that CMS regions are consistently recruited for both thinking about the psychological characteristics of oneself or others[34] and when engaging in the exact same paradigm we employed in this study; the “SRE levels-of-processing paradigm”[35], [36], [37]. However, within ASC, recent evidence points towards disruption of CMS function when mentalizing[6], [40], [41], [42], [43] and during rest[72]. The idea that CMS dysfunction is important in ASC is also corroborated by studies in individuals with high levels of alexithymia. Moriguchi and colleagues[73] found similar behavioral results to our study, in that individuals with high levels of alexithymia are also impaired at mentalizing. Individuals with high levels of alexithymia also exhibited a similar pattern of reduced activation in CMS regions that were previously found using the same mentalizing task in ASC[43]. Thus, our study converges on the idea that CMS dysfunction is paramount to the social and self-referential impairments in ASC. Future work investigating the developmental trajectory of CMS structural and functional organization in the brain is needed. Furthermore, future investigation on the development of self-referential cognition and its causal relationship with empathy may prove fruitful for informing our understanding of the neurodevelopment of ASC.
